# Ontology roadmapping and case study implementation

**DOI:** 10.2903/j.efsa.2024.e221201

**Published:** 2024-12-17

**Authors:** European Food Safety Authority (EFSA)

## Abstract

This publication is linked to the following EFSA Supporting Publications articles: http://onlinelibrary.wiley.com/doi/10.2903/sp.efsa.2024.EN-9118/full, http://onlinelibrary.wiley.com/doi/10.2903/sp.efsa.2024.EN-9119/full, http://onlinelibrary.wiley.com/doi/10.2903/sp.efsa.2024.EN-9120/full

EFSA is working to harness the potential of artificial intelligence (AI) in its operations, with the objective to become an AI‐ready organisation by 2027. Ontologies, representing a structured form of knowledge with machine‐readable definitions and inter‐relationships, were recognised as foundational to organise the vast structured and unstructured data from diverse sources, improving AI algorithms' effectiveness and maintenance. Such ontologies were deemed essential for automating and enhancing EFSA's evidence management process, with a particular emphasis on natural language processing (NLP) tasks in systematic review (SR). While EFSA has successfully implemented harmonised systems for structured data collections using consistent terminologies, the challenge lies in managing the vast amounts of unstructured data and connecting all datasets together. Ontologies can play a crucial role in bringing structure, creating links and recognising patterns within this unstructured data, enhancing interoperability and paving the way for effective AI adoption. For that purpose, a project explored the benefits of developing and adopting top‐level and domain‐specific ontologies tailored to the needs of EFSA.

The project was covered by specific contract number 01 implementing the framework contract OC/EFSA/DATA/2021/01 and so far delivered three reports reproduced in this issue. Key among them was the development of an inventory of existing food‐related ontologies, both within and outside EFSA, assessing their quality, maintenance and relevance to EFSA's work (Tzitzikas, Fafalios, Kritsotaki, et al., 2023). This inventory served as a foundation for identifying and prioritising case studies where ontologies could be applied. Eighteen case studies were identified, covering diverse applications such as standardising vocabulary for SR data extraction, automating toxicological data extraction, improving data sharing with external stakeholders, and enhancing multilingual support and synonym/typo resilience for the FoodEx2 system. The prioritised case studies were selected based on a risk/benefit analysis and their potential to include AI applications. From these, three were selected for proof of concept (POC) implementation, ensuring that at least one involved an AI component. Each case study included a detailed maintenance plan to ensure the sustainability of the implemented solutions. The other two reports propose a governance model for managing activities involving ontologies (Tzitzikas, Marketakis, Fafalios, et al., 2023) and architectural suggestions for ontology management (Tzitzikas, Marketakis, Mountantonakis, et al., 2023). Furthermore, the project facilitated the engagement and education of EFSA's personnel through an information session. This session aimed to raise awareness about the ontology project, explaining the role of ontologies in knowledge management and their importance in AI applications.

Overall, the project highlighted the strategic value of ontology management at EFSA, demonstrating the transformative potential of structured knowledge representation in enabling AI technologies to streamline and enhance EFSA's food safety operations. The project increased awareness of the potential benefits related to interoperability and adherence to FAIR principles that cannot be demonstrated through use cases based on the current EFSA processes. Given the relevance of these potential benefits, it is therefore proposed to move forward by investigating ontologies adoption opportunities with a more strategic and forward‐looking perspective involving AGoD, EC and research teams.
